# Plasmonic field confinement for separate absorption-multiplication in InGaAs nanopillar avalanche photodiodes

**DOI:** 10.1038/srep17580

**Published:** 2015-12-02

**Authors:** Alan C. Farrell, Pradeep Senanayake, Chung-Hong Hung, Georges El-Howayek, Abhejit Rajagopal, Marc Currie, Majeed M. Hayat, Diana L. Huffaker

**Affiliations:** 1 Electrical Engineering Department, University of California at Los Angeles, Los Angeles, CA 90095, USA; 2 Optical Sciences Division, Naval Research Laboratory, Washington, DC 20375, USA; 3 Center for High Technology Materials and Electrical and Computer Engineering Department, University of New Mexico, Albuquerque, New Mexico 87106, USA; 4 California NanoSystems Institute, University of California at Los Angeles, Los Angeles, CA 90095, USA

## Abstract

Avalanche photodiodes (APDs) are essential components in quantum key distribution systems and active imaging systems requiring both ultrafast response time to measure photon time of flight and high gain to detect low photon flux. The internal gain of an APD can improve system signal-to-noise ratio (SNR). Excess noise is typically kept low through the selection of material with intrinsically low excess noise, using separate-absorption-multiplication (SAM) heterostructures, or taking advantage of the dead-space effect using thin multiplication regions. In this work we demonstrate the first measurement of excess noise and gain-bandwidth product in III–V nanopillars exhibiting substantially lower excess noise factors compared to bulk and gain-bandwidth products greater than 200 GHz. The nanopillar optical antenna avalanche detector (NOAAD) architecture is utilized for spatially separating the absorption region from the avalanche region via the NOA resulting in single carrier injection without the use of a traditional SAM heterostructure.

The early theoretical work of McIntyre^1^ and Emmons^2^ showed that the excess noise and bandwidth of an avalanche photodiode is determined by the stochastic nature of the avalanche process and is a function of the ratio, *k*, of the electron, *α*, to hole, *β*, ionization coefficients. A key assumption of their work was that (1) a carrier experiences a spatially uniform electric field and (2) the multiplication region is much larger than the ionization path length, such that the excess noise at a given gain, *M*, is determined by bulk ionization coefficients. However, since their early work it has been shown that shrinking the volume of the avalanche region^3^,^4^,^5^,^6^,^7^ and engineering sharp gradients in the electric field^8^ lowers the effective ratio of the ionization coefficients, 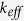, resulting in lower excess noise factors and higher gain-bandwidth products^9^,^10^ beyond what is predicted by bulk values of the ionization coefficients. The improved statistics of the impact ionization process is attributed to the ionization path length of carriers having a narrower probability distribution due to dead space. The dead-space effect can dramatically reduce the excess noise^6^, resulting in a *k*_*eff*_ that is much smaller than the intrinsic material *k*^4^,^11^,^12^. Nanopillars offer a path towards further miniaturizing the multiplication volume and exploiting the dead-space effect. Although nanopillar-based APDs have been reported^13^,^14^,^15^,^16^,^17^, all but one are single pillar demonstrations having nanoscale active areas with extremely lossy free-space or optical fiber coupling, limiting their usefulness.

In this work, we provide the first excess noise measurements on nanopillar-based APDs at 1.06 *μm* with highly localized and physically separate optical absorption and multiplication regions within a nanopillar array. The noise measurement shows a significant reduction in excess noise compared to bulk and is the first exploitation of dead space effects utilizing a 3D electric field within a nanopillar. We have also developed a novel modeling scheme for the analysis of avalanche multiplication process in the 3D electric field within a nanopillar. Recursive dead-space multiplication theory has been generalized in 3D dimensions (3D-DSMT) to analyze our experimental data and has been found to be in good agreement.

Arrays of p-doped InGaAs nanopillars with a InGaP passivation shell were grown on n+doped GaAs (111)B substrate by catalyste-free, selective-area epitaxy using metal-organic chemical vapor deposition (MOCVD). The lithographically defined patterned growth mask is systematically optimized in terms of array pitch in order to tune the enhanced optical absorption at a plasmonic resonance to the desired wavelength^18^. The detector active area was 40 μm × 40 μm, corresponding to 1156 to 2809 nanopillars for a pitch of 1150 nm to 750 nm, respectively. Figure 1a shows a tilted (30°) scanning electron micrograph of the as-grown nanopillar array. The fabricated device is shown in Fig. 1b, and the inset shows the result of the tilted deposition: a self-aligned nanohole array which functions as a 3-dimensional plasmonic antenna. Ground-signal-ground (GSG) contacts were used for noise spectral density and high-speed measurements.

The room-temperature dark current, photocurrent, and the extracted gain, *M*, as a function of bias measured from a typical device is shown in Fig. 2a. A phase-sensitive measurement was used to distinguish photocurrent from dark current. It is important to accurately determine the primary photocurrent in order to avoid overestimating the gain, thereby underestimating the excess noise factor. In typical planar APDs the primary photocurrent is determined from the DC photocurrent vs. bias characteristics. There is a clear plateau of the photocurrent with increasing bias as a result of the electric field extending through the absorption region. This plateau, or punch-through, is usually identified as the primary photocurrent. However, in the NOAAD, with increasing bias the depletion region extends through a 3-dimensional spatial distribution of photogenerated carriers. As a result, the primary photocurrent cannot be extracted from the DC photocurrent vs. bias characteristics. The primary photocurrent can be determined directly by measuring the photocurrent noise for increasing reverse bias until the measured noise begins to exceed the calculated shot noise. The photocurrent at a bias immediately before the photocurrent noise exceeds the calculated shot noise is taken as the primary photocurrent (Supplementary Figure 1). The gain is calculated by normalizing the bias dependent photocurrent to the primary photocurrent, and reaches a peak value of *M *= 96. To characterize the nanopillar optical antenna, reflectance spectromicroscopy measurements were performed and correlated to the spectral photoresponse of the NOAAD (Fig. 2b). The reflectance spectra exhibited several reflection minima at wavelengths determined by the nanopillar array pitch (Supplementary Figure 2). There is a maximum in the photoresponse at 1060 nm where there is a reflection minimum, implying enhanced absorption due to the plasmonic resonance. Indeed, FDTD simulations show a clear absorption “hot-spot” in the exposed nanopillar tip at the plasmonic resonance (Fig. 2c) that is absent for off-resonant absorption (Fig. 2d).

Although very large gain is achievable in the NOAAD, an APD’s maximum *usable* gain is that which results in the optimum signal-to-noise ratio and is determined by the excess noise factor. Minimizing the excess noise is therefore of considerable interest and is an active area of research. A unique feature of NOAAD optical absorption is the localization of optical absorption to the exposed nanopillar volume, while the avalache region is located at the nanopillar-substrate interface. Since optical absorption near the multiplication region is dramatically reduced in the NOAAD, a reduction in the excess noise as compared to double-carrier multiplication in bulk InGaAs is expected. This effect was studied through detailed modeling and simulations. Although the recurrence theory for avalanche multiplication, including the statistics of gain and impulse-response under non-uniform, static electric fields is well-known for 1D electric fields^6^,^19^, both the magnitude and direction of the electric field in the NOAAD vary in space. This is in direct contrast to planar APDs, which assume an internal electric field that only varies along the axis of the device. Here, we introduce a generalization of the recurrence theory, 3D-DSMT, to account for a 3D electric field. The electric field profile (Fig. 3a) was calculated by fitting the modeled capacitance-voltage (CV) characteristics to the measured CV (Supplementary Figure 3) by adjusting the doping profile within the nanopillar. Care has to be taken when extracting electric fields in nanostructures since the surrounding material plays a major role in fringing the electric field lines^20^. A 3D electrostatic model of a single unit cell of the NOAAD is developed taking into account both the surrounding cyclotene dielectric polymer and a cathode geometry which accounts for the metal on top of the nanopillar, on the sidewalls and on top of the cyclotene. A 2D cut through the center of the nanopillar, shown in Fig. 3b, shows the electric field radiating out from the junction area. Note that the nanopillar extends beyond the junction area due to radial overgrowth. The paths of electrons generated outside the multiplication region were numerically calculated to determine their position and energy upon entering the multiplication region (Fig. 3c). The ionization parameters were taken from measured impact ionization rates^21^.

The details of 3D-DSMT are described in the Supplementary Information. Figure 4b shows the mean gain as a function of applied bias for the electron trajectories shown in Fig. 3c. Since the gain reaches large values at a different bias voltage for each electron trajectory, it is not possible to calculate an average gain vs. bias curve. Nonetheless, by calculating the average *bias* at which the gain reaches 50, we find the calculated average bias of 6.9 V agrees very well with the measured bias of 6.8 V. The noise spectral density was measured under both dark and illuminated conditions, shown in Fig. 4b. The photocurrent noise was calculated by subtracting the dark current noise from the noise under illumination. The simulated excess noise factor is shown in Fig. 4c (solid line), along with the measured excess noise factor (symbols) and the calculated excess noise factors using McIntyre’s model (dotted lines). The excess noise calculated using 3D-DSMT is a weighted average based on the photogeneration profile. By modeling electron initiated multiplication and dead space effects, a good fit to the experimental data is achieved. The best fit to McIntyre’s model occurs for 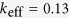, a significant reduction compared to bulk InGaAs, for which 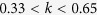 for mixed carrier injection, while 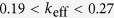 has been reported for single carrier injection^22^.

The ability to tightly confine optical absorption to the exposed nanopillar tips means that instead of following Beer’s Law, there is a sharp cut-off in the optical absorption near the cyclotene-air boundary. This allows for a thin absorption region to be located very close to the avalanche region, minimizing the transit time through the low-field drift region. The temporal response of the NOAAD to a 120 fs laser pulse was measured with a 50 GHz oscilloscope as a function of bias, shown in Fig. 5a. By normalizing the temporal response with respect to the peak value we see the rise time of 18 ps is unaffected by increasing bias, indicating the avalanche build-up time is not limiting the response time. On the other hand, the fall time clearly decreases, ranging from 297 ps to 76 ps with increasing reverse bias. For the NOAAD, the capacitance monotonically decreases as the depletion region grows with increasing reverse bias until the parasitic contact capacitance of 250 fF is reached, resulting in an RC-limited bandwidth of 2.8 GHz. The frequency response is plotted in Fig. 5b,c shows the bandwidth as a function of gain. The bandwidth increases with gain until a plateau is reached in the −3 dB bandwidth at 2.1 GHz. At the highest measured gain of *M *= 96, a gain-bandwidth product (GBP) of 201 GHz is achieved.

In summary, we have demonstrated a novel NOAAD architecture based on position-controlled arrays of III–V semiconductor nanopillars employing a self-aligned plasmonic optical antenna. The focusing of light near the exposed nanopillar tip physically separates the absorption region from the multiplication region, favoring electron injection over hole injection and reducing the excess noise compared to bulk. The measured 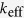 of 0.13 represents a substantial reduction in 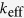 over bulk InGaAs and is the first reported excess noise measurement on a nanopillar-based APD. The NOAAD reported here demonstrate it is possible to engineer optical absorption and electric fields within nanopillars for favorable avalanche statistics. The NOAADs exhibit gain approaching 100 and a bandwidth of 2.1 GHz, for a gain-bandwidth product of over 200 GHz. We believe further optimization is possible by bringing the absorption region closer to the avalanche region, resulting in increased bandwidth with minimal effect on noise.

## Methods

### InGaAs nanopillar growth

InGaAs nanopillars were grown on a n-doped GaAs (111)B substrate by selectrive area epitaxy using metal-organic chemical vapor deposition in an Emcore vertical-flow reactor at a pressure of 60 torr and temperature of 730 °C. A passivation shell was subsequently grown *in situ* at 600 °C. The primary precursers were trimethylindium, trimethylgallium, tertiarybutylarsine, and tertiarybutylphosphorus; the p-dopant was dimethylzinc. The V/III ratio for the InGaAs core and the InGaP shell were 43 and 26, respectively. Nanopillar height and diameter showed a dependence on array pitch, with height decreasing and diameter increasing for increasing pitch. The InGaP passivation shell thickness was 10 nm.

### Device fabrication

Nanopillar arrays were planarized by spin coating with bisbenzocyclobutene (CYCLOTENE, Dow Chemical) dry etch polymer and hard cured at 250 °C for 60 minutes in a Carbolite high temperature oven. Vias for contact to the substrate were defined by photolithography and etched in an Oxford 80 Plus reactive ion etcher (RIE) with a 5:4 O_2_/CF_4_ gas mixture. Ohmic contact to the substrate was achieved by depositing germanium/nickel/germanium/gold (50 nm/100 nm/150 nm/200 nm) by electron-beam evaporation (CHA Solution) and thermal annealing at 380 °C for 30 s. The nanopillar tips were subsequently exposed using the aformentioned RIE and top contacts were defined by photolithography. The exposed nanopillar tips were electrically contacted with chrome/gold (10 nm/150 nm) deposited with the substrate mounted at an angle, resulting in a self-aligned nanohole array.

### Excess noise

The excess noise was measured by illuminating the APD with a 1064nm laser (Orbits Lightwave Ethernal SlowLight) with shot noise limited RIN and measuring the noise power spectral density with a network signal analyzer (Stanford Research Systems SR780) at 100 kHz, well above 1/*f* noise. DC bias was applied through a bias tee using a low noise power supply (Agilent B2961A) and ultra low noise filter (Agilent N1294A-021). The impedance for conversion of power spectral density to current spectral density was measured by first measuring the DC photocurent at a given optical power, then modulating the laser at 5 kHz and measuring the power spectral density at 5 kHz with the signal analyzer. Assuming the peak-to-peak current is equal to the DC current, the impedance can easily be calculated. The amplifier and dark current noise was measured and subtracted from the APD noise under illumination. Gain vs. incident power was measured to ensure gain saturation does not occur at the incident optical power used for the excess noise measurement.

### Multiplication gain

To determine the gain, the expected shot noise was calculated from current measurements at each bias and compared to the measured noise at the same bias. The primary responsivity was calculated at the bias immediately before the measured noise began to exceed the calculated shot noise. The gain was calculated by normalizing the responsivity to the primary responsivity.

### Reflectance spectromicroscopy

Optical reflectance of the NOAAD array is measured using FTIR micro spectroscopy system configured using a Thermo Scientific Nicolet Continuum microscope and a Thermo Scientific Nicolet 8700 spectrometer. The spectrometer utilizes a CaF2 beamsplitter, while the microscope is equipped with an internal MCT-a detector to increase signal to noise ratio.

### Temporal response

The temporal response was generated by illuminating the APD with a Coherent Ti-Sapphire laser with a 120-fs pulse width and 76-MHz repetition rate tuned to a 998-nm center wavelength. Transient pulse characteristics were measured with a microwave probe (Cascade ACP40-GSG-150) and a 50-GHz oscilloscope (Tektronix SD-32 sampling head). A microwave bias-T (Wiltron V250) was used to bias to the sample while passing the transient signal to the oscilloscope. The bandwidth was extracted from the temporal response by FFT analysis.

## Additional Information

**How to cite this article**: Farrell, A. C. *et al*. Plasmonic field confinement for separate absorption-multiplication in InGaAs nanopillar avalanche photodiodes. *Sci. Rep*. **5**, 17580; doi: 10.1038/srep17580 (2015).

## Supplementary Material

Supplementary Information

## Figures and Tables

**Figure 1:**
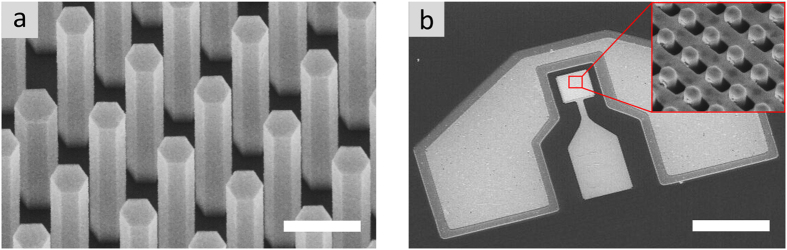
NP array and device fabrication. (**a**) Tilted SEM of as-grown InGaAs nanopillar array. Scale bar, 600 nm. (**b**) Fabricated NOAAD with ground-signal-ground (GSG) contacts for high speed measurements. The signal contact is deposited directly on the BCB to electrically isolate it from the substrate. Scale bar, 80 μm. Inset: Tilted metal deposition results in a self-aligned nanohole array.

**Figure 2:**
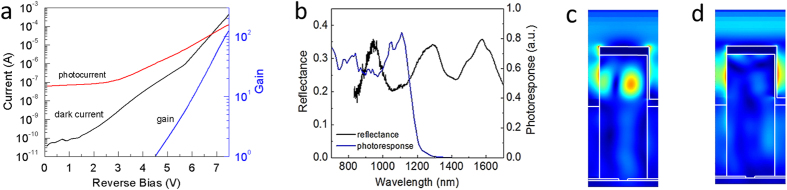
Electrical and optical characterization. **(a)** Measured dark current and photocurrent at room temperature of an APD with a 40 μm X 40 μm active area. The extracted multiplication gain approaches 100. (**b**) Reflectance spectrum and spectral photoresponse exhibit a minimum and maximum, respectively, at 1060 nm. (**c**) Photogeneration within the nanopillar at a plasmonic resonance and (**d**) off resonance.

**Figure 3:**
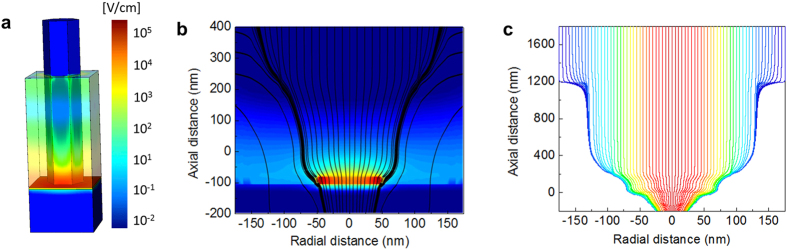
Electric field modeling. (**a**) 3D electric field distribution within nanopillar [V/cm]. (**b**) Electric field lines, drawn in black, show a field that radiates out from the junction area throughout the nanopillar (the nanopillar extends beyond the junction area due to radial overgrowth). (**c**) The electron trajectories through a nanopillar are numerically calculated showing that electrons generated in the nanopillar tip are funneled to the multiplication region by the 3D electric field.

**Figure 4:**
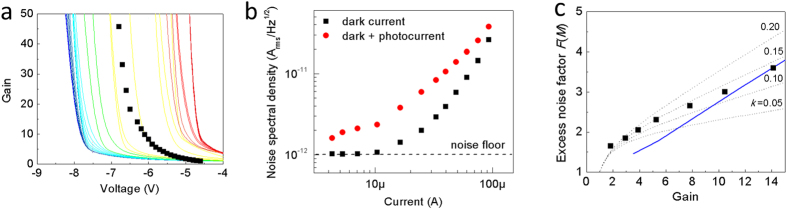
Excess noise. (**a)** The gain is calculated for each electron trajectory in Fig. 3c and matches well with the measured gain (symbols). (**b)** Measured current noise spectral density of the dark current and dark current plus photocurrent. Note that the photocurrent noise could not be measured at the highest gains due to the increase in dark current noise. **(c)** Excess noise vs. Gain of the InGaAs NOAAD. The symbols show the measured excess noise factor and the solid line is calculated using 3D-DSMT. The dotted lines are calculated using Mcyntre’s theory for (from bottom to top) *k *= 0.05, 0.10, 0.15 and 0.20.

**Figure 5:**
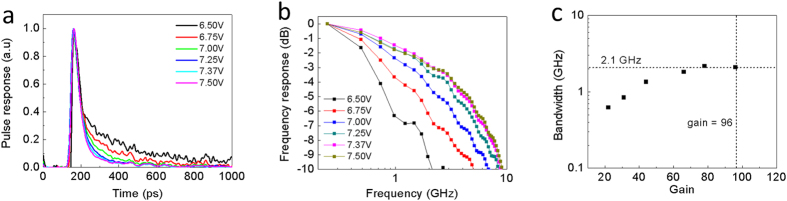
High speed measurements. (**a)** Normalized temporal response of NOAAD measured with 50 GHz oscilloscope and coherent Ti-Sapphire laser (998 nm, 120 fs pulse, 76 MHz repetition rate). Only the fall time is affected by increasing reverse bias. (**b)** Extracted frequency response. (**c)** Measured −3 dB bandwidth versus gain.
