# Supplementary material for: Plasmonic field confinement for separate absorption-multiplication in InGaAs nanopillar avalanche photodiodes
Source: Sci Rep. 2015 Dec 2;5:17580. doi: 10.1038/srep17580 (PMC4667247; doi:10.1038/srep17580)
Supplement: Supplementary Information [file srep17580-s1.pdf]

Plasmonic field confinement for separate absorption-multiplication in InGaAs nanopillar avalanche photodiodes

Alan C. Farrell[1a], Pradeep Senanayake[1a*], Chung-Hong Hung[1], Georges El-Howayek[3], Abhejit Rajagopal[1], Marc Currie[2], Majeed M. Hayat[3] & Diana L. Huffaker[1,4]

[1] Electrical Engineering Department, University of California at Los Angeles, Los Angeles, CA 90095, USA
[2] Optical Sciences Division, Naval Research Laboratory, Washington, DC 20375, USA
[3] Center for High Technology Materials and Electrical and Computer Engineering Department, University of New Mexico, Albuquerque, New Mexico 87106, USA
[4] California NanoSystems Institute, University of California at Los Angeles, Los Angeles, CA 90095, USA
[a] These authors contributed equally to this work.
*Corresponding author email: p.senanayake@ucla.edu

SUPPLEMENTARY INFORMATION

Excess noise measurements

In order to convert the measured power spectral density to current spectral density, the system impedance must be known. The impedance was measured as follows: The DC photocurrent, I_{DC}, of an InGaAs PIN photodiode was measured at a given incident laser power using a standard semiconductor parameter analyzer. The laser was then mechanically chopped at 5 kHz and the peak-to-peak power, P_{peak}, at 5 kHz was measured using the signal analyzer. Assuming the peak-to-peak current produced by the photodiode is equal to the DC photocurrent (which is reasonable, given the very low modulation frequency), then $I_{peak} = I_{DC}$ and $P_{peak} = I_{peak}^2 R$, where R includes the input impedance of the signal analyzer. Therefore, $R = P_{peak}/I_{peak}^2$.

The noise measurement setup was then tested by measuring the noise power of an InGaAs PIN photodiode (at zero bias) by varying the incident laser power, shown in Fig. 1 for two different transimpedance gain settings (after 500 averages). The measured noise (symbols) was converted to current spectral density using the measured value of R. The laser RIN line is also plotted (calculated from manufacturer specifications). The pre-amp noise floor was measured. The red dashed line is the total noise calculated from the pre-amp noise floor, the shot noise, and the laser RIN.

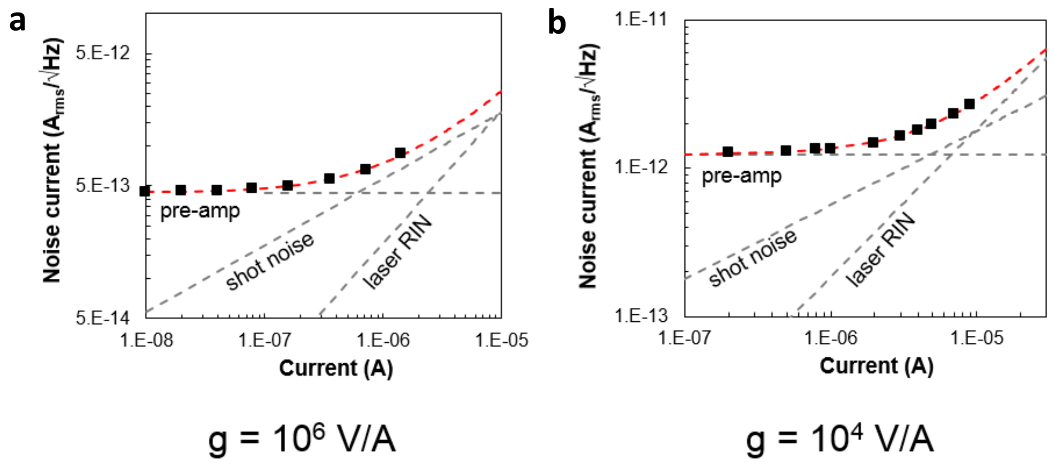

Figure 1. Measured noise (symbols) of an InGaAs PIN photodiode along with the various noise sources: pre-amp, laser RIN, and shot noise (grey dashed lines). The total noise is shown in the red dashed line. Transimpedance gains of a) 10^6 V/A and b) 10^4 V/A were tested to ensure the measured impedance is independent of pre-amp gain settings.

Subtracting the pre-amp noise and laser RIN from the measured noise gives the shot noise, shown in Fig. 2. By carefully accounting for all the noise sources in the measurement setup and measuring the system impedance, a very accurate measurement of shot noise can be performed.

Since a PIN photodiode at zero bias was used for the calibration of the noise measurement setup, there is no uncertainty about the M = 1 reference point because there is no gain. The InGaAs PIN was then replaced by the nanopillar APD to perform excess noise measurements.

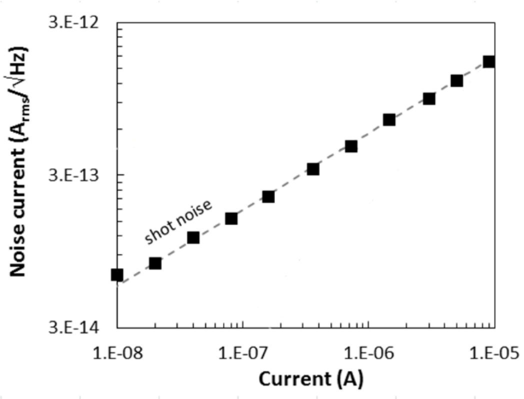

Figure 2. Noise calculated by subtracting the pre-amp noise and the laser RIN from the total measured noise under illumination. The grey dashed line is the calculated shot noise.

To accurately determine unity gain photocurrent, noise spectral density measurements on the photocurrent were performed. Fig. 3 shows the noise measurement with increasing applied reverse bias. At low biases the noise of the NOAAD follows the expected shot noise up to 10 µA of photocurrent which corresponds to the unity gain photocurrent. Further increasing the bias results in the noise to be dominated by excess noise.

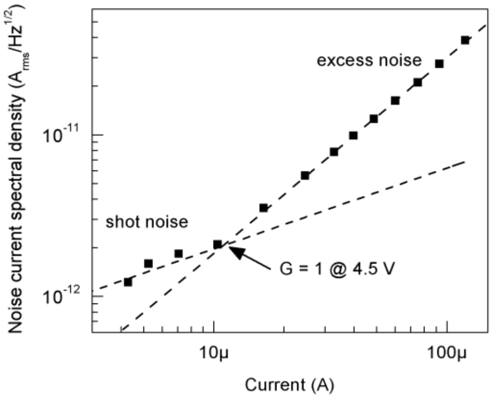

Figure 3. Measured noise for increasing bias. The unity gain point is taken at the point immediately before the noise exceeds the calculated shot noise.

Gain saturation

In order to ensure gain saturation does not occur during the excess noise measurement, photocurrent measurements were performed at various incident optical powers. The gain is then plotted as a function of incident power at a bias of 4.7 V to 6.2 V (Fig. 4). As the bias is increased the gain saturation effect becomes more prominent at high optical power. Therefore, the optical power used for excess noise measurements was kept below 30 μW.

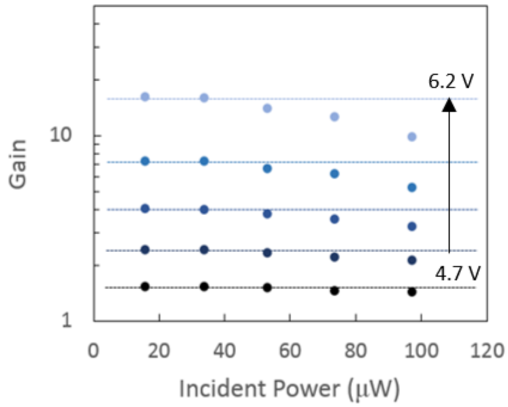

Figure 4. Gain as a function of incident optical power for biases of 4.7 V to 6.2 V.

Nanopillar Optical Antenna

Resonant absorption wavelength of the (NOA) was determined by FTIR reflection spectromicroscopy. Continuum microscope equipped with a 10x reflective objective with an aperture under filling the sample was used to quantify reflection. Fig. 5 shows that optical absorption due to the Nanopillar Optical Antenna results in reflection dips resulting in ~ 10% of reflected light on resonance.

Capacitance Measurement

The capacitance of a single nanopillar was estimated by measuring the CV of the NOAAD as well as the CV of a dummy device (identical to the NOAAD, but without nanopillars, fabricated on the same sample). The CV of the dummy device is independent of bias, since there is no contribution from junction capacitance from the nanopillars. Since the voltage on the contact is equal to the voltage on the nanopillars, we treat the system as two parallel capacitors and simply subtract the dummy capacitance from the capacitance of the NOAAD. Finally, we once again assume that the array of nanopillars is connected in parallel and divide by the number of nanopillars to get the capacitance of a single nanopillar.

3D-DSMT

The traditional recursive methods of studying the avalanche process within the multiplication region developed by Hayat et al.assume planar APDs where the direction of the internal electric field does not change spatially[11,26]. That is, the derivations were performed under the assumption that the electric field was one-dimensional in nature. Since the electric field for a NOADD has components in 3 orthogonal directions, it must be represented by a vector function that depends on the 3-dimensional position within the multiplication region: $\mathbf{E}(x; y; z)$. The 3D electric field was determined by fitting the experimental capacitance-voltage characteristic for a single unit cell of the NOAAD, shown in Fig. 6, using a 3D drift-diffusion model developed in Sentaurus as shown in Fig. 3 of the main text. The 3D contact of the NOAAD and surrounding BCB dielectric was taken into account in the electrostatic model.

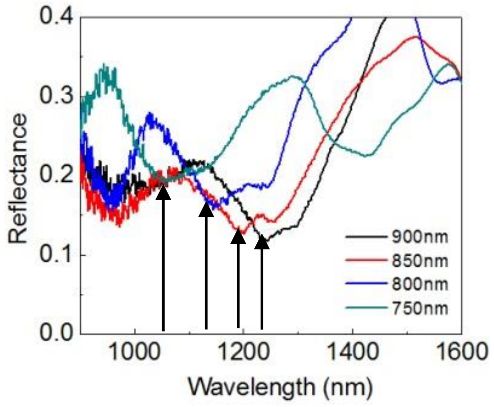

Figure 5. Reflectance spectromicroscopy measurements on arrays with varying pitch show a clear dependence of the reflection minima on the array pitch.

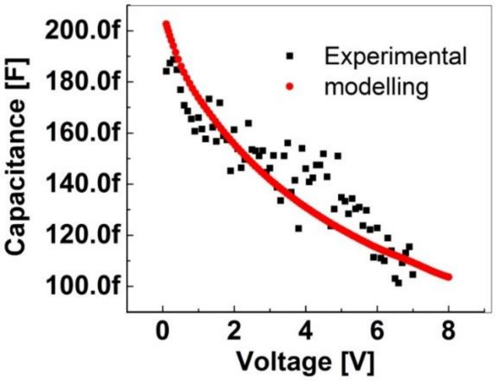

Figure 6. Measured capacitance-voltage (CV) characteristics for unit cell of and the modeled CV used to extract the doping profile within a nanopillar. A 40 X 40 array of nanopillars produced a capacitance well above the parasitic capacitance and the noise floor of the measurement system.

After determining all the possible paths of the carriers in the NOADD 3-dimensional multiplication region, we apply this generalized 3D-DSMT to all the possible trajectories. Each of these potential paths has its own individual probability of occurring; that is, depending on the entrance position of a photocarrier to the multiplication region, an avalanche process occurs at a specific path. We calculated the mean gain and excess noise for each trajectory, triggered by an electron injected at the beginning of the multiplication region. Since these electrons originate from photon absorption within the plasmonically enhanced nanopillar, we can determine the entrance probability using photon absorption rates and the electric field within the absorption region. Then the average gain and overall excess noise factor are found by averaging over all possible paths.

The electron and hole saturation velocities are assumed as $6.7 \times 10^6\ cm/s$. The ionization parameters for InGaAs are given in Table 1. Note that the ionization coefficients reported by Ng, *et al.*, were tested as well but did not accurately predict the IV characteristics of our device an so the coefficients of Pearsall were used.

Carrier	A $(cm1)$	B (V/cm)	m	Threshold Energy (eV)
Electrons	1.80×10^7	1.95×10^6	1	1.20
Holes	2.56×10^7	2.20×10^6	1	1.00

Table I: Ionization Parameters for InGaAs
